# Leukocytes: The Double-Edged Sword in Fibrosis

**DOI:** 10.1155/2015/652035

**Published:** 2015-10-19

**Authors:** Jakub Kryczka, Joanna Boncela

**Affiliations:** Institute of Medical Biology, Polish Academy of Sciences, 93-232 Lodz, Poland

## Abstract

Skin tissue scar formation and fibrosis are often characterized by the increased production and deposition of extracellular matrix components, accompanied by the accumulation of a vast number of myofibroblasts. Scaring is strongly associated with inflammation and wound healing to regain tissue integrity in response to skin tissue injury. However, increased and uncontrolled inflammation, repetitive injury, and individual predisposition might lead to fibrosis, a severe disorder resulting in the formation of dense and stiff tissue that loses the physical properties and physiological functions of normal tissue. Fibrosis is an extremely complicated and multistage process in which bone marrow-derived leukocytes act as both pro- and antifibrotic agents, and therefore, few, if any, effective therapies are available for the most severe and lethal forms of fibrosis. Herein, we discuss the current knowledge on the multidimensional impact of leukocytes on the induction of fibrosis, focusing on skin fibrosis.

## 1. Introduction

Skin tissue integrity is a crucial factor to maintain the homeostasis generated through physical barriers, separating the organism from the environment. Every disruption of dermal integrity triggers a complicated cascade of events, including rapid blood clot formation, inflammatory response, and wound healing, leading to the restoration of the integrity and formation of new tissue. However, repaired structures, known as scars, are nonfunctioning, tight, and tense masses of fibrotic tissue that maintain 70–80% of normal strength, with even less flexibility [1]. Inflammatory responses are necessary for wound healing, preventing multiple infection and contamination and stimulating the proliferation, revascularization, and remodeling of the extracellular matrix [2, 3]. Nevertheless, wound healing might become uncontrolled and, combined with the inflammatory response, results in massive fibrotic tissue formation called fibrosis. In this review, we will focus on the molecular mechanisms underlying skin fibrosis as a post-wound-healing pathological disorder and the impact of bone marrow-derived cells and inflammation on the formation of scars.

## 2. Fibrosis and Wound Healing: Two Faces of the Same Story

Fibrosis is a pathological process that occurs in many different organs (organ specific fibrosis), such as skin, kidney, heart, lung, and liver [4], which might also take the form of systemic sclerosis (SSc), a global, progressive, and autoimmune disorder, characterized by an extremely poor prognosis and high mortality [5, 6]. According to the United States government, every year, in the USA, around 45% of natural deaths can be associated with different fibrotic disorders [7]. Although the etiology and triggering cascade might differ, fibrosis is characterized by the increased production and deposition of extracellular matrix (ECM) components, including collagen type I, fibronectin, hyaluronan, and elastin, and the accumulation of activated, *α*SMA-positive, and collagen-secreting fibroblasts, called myofibroblasts [4, 8, 9]. Myofibroblasts exhibit the ultimate fibroblast phenotype. Many authors refer to fibrosis in the context of “uncontrolled” or exceeded wound healing, as an effect of long-term inflammation or mechanical irritation [10].

**Figure 1:**
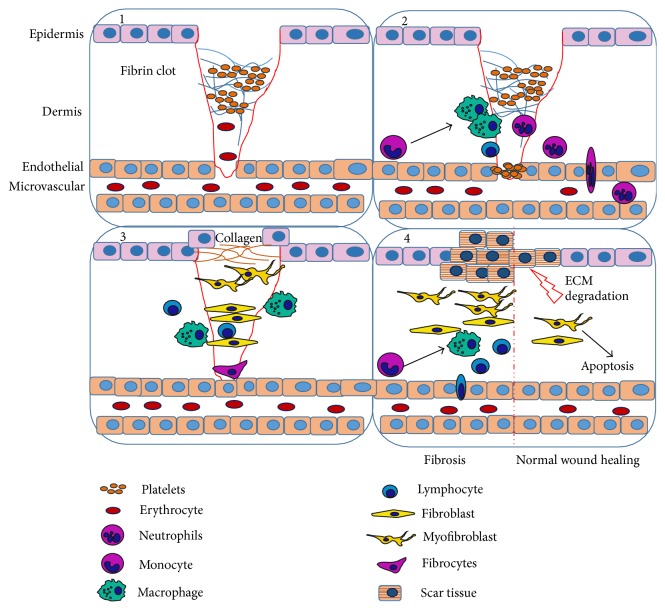
The stages of wound healing. 1, coagulation: after injury, fibrin clot is formed. Trapped platelets degranulate and release inflammatory chemokines. 2, inflammation: leukocytes enter wound site. Neutrophils appear first, followed by macrophages and lymphocytes. Leukocytes clear wound from bacteria and any foreign bodies, respectively, recruiting fibroblasts. 3, proliferation: activated fibroblasts, myofibroblasts, produce and deposit ECM components that serve as skeleton during tissue regeneration process. 4, remodeling, the final stage in normal wound healing: excess amount of ECM is degraded, fibroblasts and myofibroblasts undergo apoptosis, and inflammatory cells leave regenerated tissue. However, during fibrosis, inflammation is prolonged and ECM deposition is highly increased by myofibroblasts.

Wound healing is extremely complex and involves the cooperation of many cell types. This process can be divided into four overlapping phases: coagulation, inflammation, proliferation, and remodeling. Skin injury results, inter alia, in the disruption of endothelial and epithelial cells integrity [10]. Damaged cells release inflammatory mediators that trigger the coagulation cascade, platelet recruitment, and blood clot formation. Degranulated and activated platelets present in the blood clot release multiple chemokines and growth factors (TGF-*β*1, PDGF), which recruit inflammatory cells. Neutrophils appear first, followed by macrophages and lymphocytes. Platelets also participate in the chemotaxis and recruitment of fibroblasts and endothelial cells [11–13]. The first two phases are often treated as one phase, representing the inflammatory stage. The blood clot comprises cross-linked fibrin and extracellular matrix proteins, such as fibronectin, vitronectin, and thrombospondin. This structure serves as a physical barrier that closes the blood vessel, a reservoir of growth factors, and a matrix on which regenerated tissue is formed [12, 14]. The next phase, proliferation, results from hypoxic conditions and reactive nitrogen species (RNS) production from macrophages [15, 16]. During this phase, angiogenesis occurs, forming new capillaries and facilitating the delivery of nutrients to the wound. In addition to nutrients, collagen-secreting myofibroblasts are recruited to the wound microenvironment [17]. As previously described, myofibroblasts are activated, *α*SMA-positive, and collagen-secreting fibroblasts that deposit new ECM components, primarily fibronectin and collagen type I, to replace the clot-formed matrix, often forming a scar [10, 18]. In physiological wound healing, remodeling is the final phase. During this phase, myofibroblasts and some vascular cells undergo apoptosis and disappear from the regenerated microenvironment [19]. Moreover, the synthesis of ECM components is reduced but not fully terminated [11], and remodeling is primarily regulated through different matrix metalloproteinases (MMPs) and their inhibitors (tissue inhibitor of metalloproteinases, TIMPs). After the degradation of the overexpressed ECM components, scarring is reduced and an equilibrium between synthesis and catabolism is reached [13, 20]. In fibrosis, the proliferation and remodeling phases have become pathological. Myofibroblasts constantly produce ECM components, disrupting the delicate equilibrium. The increased deposition of collagen type I and fibronectin stiffens and damages the surrounding tissue. In addition, connective tissue cells replace the original cells, creating a scar that in some cases might take an extremely severe form [13, 21, 22]. The wound healing process is shown in Figure 1.

Activated fibroblasts/myofibroblasts accumulate in the fibrotic tissue environment by three different, simultaneous mechanisms. First, these cells are derived from preexisting fibroblasts in the affected tissue through activation due to specific, profibrotic, and proproliferative mediators released from infiltrating inflammatory cells, such as T cells [23–25]. Second, myofibroblasts are recruited through bone marrow-derived fibroblast resembling cells, such as fibrocytes, CD45 and CD34 positive cells [26]. Fibrocytes transmigrate to the fibrotic environment, and in a TGF-*β*1-controlled process, these cells undergo transdifferentiation into myofibroblasts [27]. Finally, fibroblasts/myofibroblasts accumulate through the transition from endothelial or epithelial cells to mesenchymal fibroblast-like cells [5, 11].

## 3. The Endothelial and Epithelial to Mesenchymal Transition as a Key Factor in Fibrosis

During the endothelial and/or epithelial to mesenchymal transition (EndMT and EMT, resp.), cells lose their origin markers, polarity, and cell-cell connections and gain promigratory phenotypes and mesenchymal markers [28–30]. Both EndMT and EMT are physiological processes that occur during embryonic organogenesis and wound healing. Epithelial and endothelial cells establish close cell-cell contacts with a certain cell polarity, forming a solid barrier that maintains homeostasis. This barrier is formed through desmosomes and tight and adherent junctions [28, 31]. In contrast, mesenchymal cells are spindle-shaped solitary cells, possessing migratory and ECM remodeling abilities. These cells produce and secrete ECM components, such as collagen type I and fibronectin [13, 32]. During tissue development or regeneration, tightly connected cells cannot undergo migration. Therefore, after undergoing EndMT/EMT, these cells gain the migratory abilities of fibroblasts, facilitating the recruitment of these cells to certain locations. Cells do not typically undergo full transitions, often terminating in intermediate phenotypes between endothelial or epithelial and mesenchymal, and maintaining some cell-cell contacts to perform group migration rather than single cell migration [28, 33]. The endothelial to mesenchymal transition was first observed and described as the leaking and proliferation of endothelial cells during the development of chick and rat endocardial cushions (cardiac mesenchyme) [34]. EndMT and EMT are involved in pathological disorders as well. EndMT is closely associated with dermal, renal, cardiac, pulmonary, intestinal, and cystic fibrosis through the establishment of fibroblasts and myofibroblasts [35, 36]. EMT is reversible, and fibroblasts might regain epithelial phenotypes (mesenchymal to epithelial transition, MET), whereas EndMT reversibility is not well understood. The reversal of EndMT (mesenchymal to endothelial transition) has been recently observed in cardiac fibroblasts that rapidly adopt an endothelial-cell-like phenotype after acute ischemic cardiac injury [37]. However, more additional evidence suggests that EndMT is irreversible, and transformed cells cannot regain endothelial phenotypes, even after the removal of EndMT inducing factors [38]. Therefore, EndMT, which is not terminated at a certain time, could lead to the accumulation of collagen type I secreted from myofibroblasts and the irreversible transformation into fibrotic tissue [39].

Both EndMT and EMT are regulated through the zinc finger transcription factor Snail family (Snail1, Snail2, and Snail3). Snail1 is the first and most crucial transcription factor activated during mesenchymal transition. After activation, on the molecular level, Snail1 stabilizes the quantity of Twist1 transcription factor, and in cooperation, both of these proteins upregulate* ZEB1* gene expression [40, 41]. As a repressor, Snail proteins downregulate the expression of genes encoding junction proteins, such as claudin, occludin, E-cadherin (in epithelial cells), VE-cadherin, and PECAM1 (in endothelial cells). It is not clear whether Snail upregulates the genes encoding mesenchymal markers, as observed in the upregulation of myosin Va in some highly metastatic cancer cell lines, such as human lung carcinoma cell lines (A549, PG, and Calu6), human colon cancer cell lines (Lovo and SW480), human breast cancer cell lines (BICR-H1 and MCF7), and prostate cancer cell lines with the same genetic background (PG3M-1E8 and PG3M-2B4) [42], or represses epithelial/endothelial genes and therefore indirectly upregulates mesenchymal markers. Nevertheless, mesenchymal cell proteins, such as vimentin, fibronectin, collagen type I, *α*SMA, SM22*α* (transgelin), N-cadherin, calponin, and FSP-1 (fibroblast specific protein 1), are expressed during and after the transition [43–46]. The microRNA profile also changes during mesenchymal transition, revealing the significant upregulation of miR-125, Let-7c, Let7g, miR21, miR30b, and miR195 and downregulation of miR122a, miR127, miR196, and miR375 [47]. A previous study reported that the accumulation of Snail in colorectal cancer cells and in mice utricle sensory epithelia cells, after blocking the degradation of this protein through the glycogen synthase kinase-3 (GSK-3), via lithium chloride treatment or the overexpression of Snail, might trigger the transition into mesenchymal-like cells [43, 48, 49]. However, this transition is typically induced through a variety of proinflammatory cytokines and growth factors secreted from leukocytes, which act synergistically. The most important proinflammatory/profibrotic molecules are transforming growth factors *β*-1 and *β*-2 (TGF-*β*1 and TGF-*β*2), tumor necrosis factor-*α* (TNF-*α*), interleukins IL-1*β*, IL-6, IL-8, and IL-11, and fibroblast growth factor-2 (FGF-2) [38, 39, 50–54]. It has been suggested that TGF-*β* receptor is essential for mesenchymal transition signal transduction, and the overexpression of Snail might be an insufficient factor. The inhibition of TGF-*β* receptor accompanied by simultaneous upregulation of Snail does not lead to EndMT in mouse embryonic stem cell-derived endothelial cells (MESECs) [44]. However, the upregulation of the transcription factor Snail directly upregulates profibrotic and proinflammatory cytokines, such as IL-8 [55].

The secretion of TGF-*β* into the fibrotic microenvironment during inflammation is the most important Snail inducer. Snail expression might be triggered through many pathways. The most common pathway is the activation of the Smad2/3 complex. However, studies on skin cancer formation have shown that Smad2 inhibits EndMT, whereas Smad3 acts as an activator [56]. The binding of TGF-*β* to TGF-*β* receptor type II (T*β*RII) triggers heterodimerization through the activation of the TGF-*β* receptor type I kinase (T*β*RI), which activates activin-like kinase 5 (ALK5) and transduces a signal through the Smad2/3 complex with Smad4, which activates the expression of Snail [53]. TGF-*β*2 activates Smad2/3 via ALK2. The inhibition of either ALK5 or ALK2 results in the inhibition of EndMT [29, 57]. TGF-*β* also activates Snail in a non-Smad pathway, involving Wnt and Noch, via the sequestration of GSK-3 and Akt2, through the transcriptional repression of the miR-200 superfamily and the activation of the inflammatory transcription factor NF*κ*B [30, 46, 50, 58].

## 4. Leukocytes in Fibrosis: Unanswered Questions

As previously discussed, chronic inflammation is one of the main factors triggering fibrosis, particularly EndMT-based fibrosis, as EndMT is an irreversible process. Constant inflammation leads to the production of a variety of proinflammatory cytokines and growth factors secreted from different leukocytes present in the fibrotic microenvironment. However, fibrosis formation is a multidimensional and multistage process that not only involves EndMT. Leukocyte recruitment triggers many different mechanisms and pathways that might lead to disordered wound healing, myofibroblasts and collagen type 1 accumulation, scarring, and fibrosis.

### 4.1. Neutrophils

Neutrophils appear first at the site of the wound. The recruitment of these cells is initiated immediately after activated platelets degranulate and release TGF-*β*1 and PDGF. TNF-*α*, IL-1, and IL-8 released from endothelial cells also stimulate neutrophil recruitment, leading to selectin-mediated rolling adhesion towards the chemoattractant gradient. In the next phase, tight adhesion to endothelial cells occurs via integrin *β*2, followed by transmigration through the endothelial tissue. When necessary, neutrophils cross the ECM barrier along fibroblasts and transmigrate through epithelial cells to enter the wound [59]. Neutrophils begin phagocytosing invading bacteria and damaged necrotic cells to clear the wound, preparing it for the regeneration of homeostasis through scar formation. However, fetal wounds heal without scar formation, and fetal neutrophils are physiologically distinct from adult neutrophils, as these cells are less adept than adult cells, producing less cytokines and presenting lower contributions to the inflammatory response [60, 61]. Neutrophil serine protease, elastase, is secreted into the microenvironment, increasing IL-8 expression in the surrounding cells [62]. IL-8 not only is responsible for leukocyte recruitment but also might trigger EndMT and increase the survival and proliferation of endothelial-derived fibroblasts/myofibroblasts, leading to fibrosis [6, 63]. Additionally, elastase is also believed to cleave the IL-8 receptor CXCR1, interfering with neutrophil functions and antibacterial abilities, thereby prolonging inflammation, which in turn increases additional fibrosis-based changes [64]. Prolonged inflammation might also occur through the elastase-mediated degradation of complement, releasing the strong neutrophil chemoattractant, C5a [65]. Moreover, neutrophil derived oxidative burst, leading to the formation of HOCl^*∗*^ from H_2_O_2_ catalyzed through myeloperoxidase, induces injury to epithelial cells, thereby implicating the switch to fibrotic tissue deposition [66, 67]. Two different populations of neutrophils have been observed to enter the wound in mice after the induction of acute inflammation: one population has a proinflammatory function, and the second population is responsible for anti-inflammatory responses. These cells differ in size, granularity, and the expression of CD11b and Ly6G [68]. Respectively, the anti-inflammatory neutrophil response is strongly associated with the secretion of the anti-inflammatory cytokine IL-10 [69]. Moreover, a certain population of mature neutrophils, characterized as CD11c^bright^/CD62L^dim^/CD11b^bright^/CD16^bright^, have been reported to suppress T cell proliferation via the expression of the integrin Mac-1 (*α*M*β*2) [70].

The impact of neutrophils on fibrosis has been observed in pulmonary fibrotic disorders, as these cells transmigrate to pulmonary fluids (such as bronchoalveolar lavage fluid) and recruit other leukocytes [71, 72]. However, only a few clinical studies have successfully established anti-inflammatory strategies in patients with pulmonary fibrosis. Inhibition of neutrophil derived elastase as strategy of downregulation of self-destructive process of neutrophil derived protease activity as well as elastase derived IL-8 expression is currently being elucidated and brings more questions than answers. Clinically useful concepts have only just started to evolve and bring promising, but not yet convincing, answers [59, 73].

### 4.2. Macrophages

**Figure 2:**
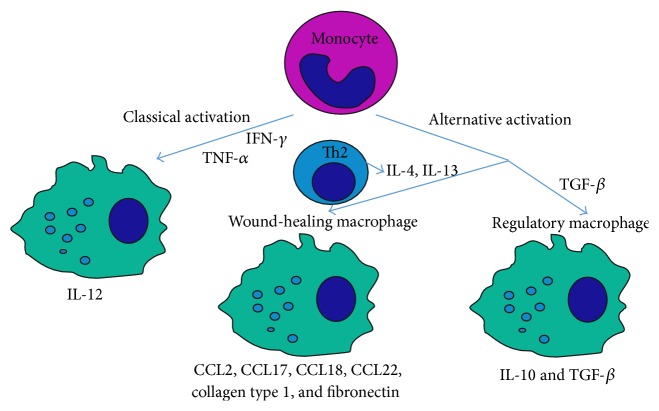
The divergent macrophage activation pathway. Macrophage activation and differentiation from monocyte in the wounded tissue depends on chemokine and growth factors availability. Two macrophage activation pathways might be distinguished, classical activation (M1) depending on interferon gamma (IFN-*γ*) and tumor necrosis factor-*α* (TNF-*α*) and alternative activation pathway (M2). Alternative activation is divided into two separate macrophage populations, IL-4 and IL-13 derived wound-healing macrophage population and TGF-*β* derived regulatory macrophage population. Despite monocyte origin, different activation pathway results in production and secretion of different chemokines and proteins into wounded tissue.

Macrophages appear as the second type of bone marrow-derived cells invading the wound site, and three to five days after injury, they become the dominant leukocyte type [10]. Monocytes, recruited through PDGF, undergo differentiation towards macrophages. Similar to neutrophils, different populations of macrophages have been reported, depending on the activation path through different chemokines and growth factors, as shown in Figure 2. Classical macrophage activation, or M1, is obtained, in particular, through the combination of interferon gamma (IFN-*γ*) and tumor necrosis factor-*α* (TNF-*α*) signaling pathways. Classically activated macrophages produce proinflammatory cytokines, including interleukin-12 (IL-12) [74]. Alternative activation, or M2, is far more complex, leading to the formation of regulatory and wound-healing macrophages. Regulatory macrophages release anti-inflammatory cytokines IL-10 and TGF-*β*, which downregulate inflammation, and also lead to the endothelial to mesenchymal transition and increase the fibroblast number at the wound site [75]. TGF-*β* pro- and anti-inflammatory roles are often described as paradox. Its abilities might shift, depending on other cytokines availability and cell type [76, 77]. It was shown that TGF-*β* administered to animals with infection or inflammation reduces severity of disease and production of proinflammatory IL-1 and TNF [78]. A second group of M2 macrophages, wound-healing macrophages, are derived through IL-4 induction. These cells secrete CC chemokine ligands, including CCL2, CCL17, CCL18, and CCL22 [72]. Wound-healing macrophages are extremely profibrotic, as these cells produce high levels of fibronectin and through CCL18 activation promote collagen production from fibroblasts/myofibroblasts [79]. Moreover, arginase activation in M2 macrophages, stimulated through IL-4, leads to the conversion of arginine to ornithine, a precursor of collagen [74, 80]. Blocking IL-4 with specific antibodies significantly decreases wound-healing, macrophage accumulation, and fibrosis formation [81].

Recent studies showed that overexpression of MMP9 in macrophage might attenuate bleomycin induced pulmonary fibrosis [82]. Respectively, production of MMP13 by Kuppfer cells was shown to be sufficient in preventing pig serum-induced rat liver fibrosis [83]. These data suggest that high level of MMPs might play key role in fibrosis reversibility. Macrophages are the main sources of MMPs that facilitate ECM degradation during remodeling phase in wound healing process; they also phagocytose apoptotic myofibroblasts and cellular debris preventing advance in the fibrotic process. However, some authors suggest strong profibrotic role of macrophage derived MMP13, as they observed that liver fibrosis was suppressed, along with fibrotic markers and inflammatory mediator expression, in MMP13-deficient mice during cholestasis-induced liver fibrosis [84]. Surpassingly, prolonging inflammation and recruitment of activated macrophages might be involved in fibrosis reversing process, as accumulating evidence strongly correlates macrophages and the macrophages derived MMPs (MMP1, MMP2, MMP8, MMP9, and MMP13) with this process [85]. Nevertheless, the role of MMP13 remains unanswered.

### 4.3. Lymphocytes

Lymphocytes recruited to injured tissue are activated through various antigens. After arrival to the wound site, these cells produce lymphokines, which in turn activate other inflammatory cells, such as macrophages [14]. Among all lymphocyte subpopulations, Th1 and Th2 are most relevant for tissue fibrosis. Th1 and Th2 lymphocytes contribute different responses to wounded tissue. Th1 acts as an antifibrotic, releasing IL-10, and Th2 acts as a profibrotic. Studies using mouse models have shown that the polarized Th2 response leads to massive collagen deposition and increased fibrosis formation. However, the Th1 response activates the genes responsible for apoptosis and acute-phase reactions [86, 87]. Among all cytokines released from Th2, the two most important and most profibrotic cytokines are IL-4 and IL-13. Both IL-4 and IL-13 share functional similarities, as these molecules transduce signal via the IL-4R/Stat6 pathway [88–90]. As previously described, IL-4 activates M2 wound-healing macrophages, resulting in collagen production and deposition. Moreover, IL-4 stimulates in the dose-dependent manner collagen synthesis in fibroblasts and is two times more effective than TGF-*β* [91]. The scleroderma mouse model (tight-skin mutant mouse Tsk/+) presented extremely increased dermal collagen expression, secretion, and deposition correlated with IL-4. Treatment with an anti-IL-4 antibody resulted in collagen downregulation and provided less fibrosis-based pathological changes [92]. The Th2-mediated secretion of IL-4 and IL-13 enhanced fibrocyte differentiation from CD14-positive precursors, thereby leading to increased fibroblast recruitment and potential fibrosis [93].

The impact of Th2-derived IL-5 on fibrosis is strongly associated with the recruitment and activation of eosinophils. Activated eosinophils secrete inflammatory factors, such as IL-13 and TGF-*β*1, into wounded tissue, resulting in fibrosis development, as shown for dermal fibrosis in a mouse model of skin allograft rejection [94]. Both IL-13 and TGF-*β*1 might induce collagen secretion from fibroblasts present in the wound; however, TGF-*β*1 is strongly associated with EndMT- and EMT-based fibrosis [95, 96].

As discussed above, Th1 and Th2 lymphocytes affect differently fibrotic tissue. Recently, it was shown that patients with cystic fibrosis and* P. aeruginosa* infection present an age-dependent dysregulation of lymphocyte T response that shifts towards Th2 lymphocyte, resulting in enhanced fibrotic tissue deposition. However, precise regulatory immune mechanism remains poorly understood [97].

### 4.4. Fibrocytes

Fibrocytes are circulating, bone marrow-derived cells that exhibit mesenchymal phenotypes. As previously described, these cells are both CD45- and CD34-positive cells that transdifferentiate into myofibroblasts. The name fibrocyte represents a combination of shared features of these cells: fibroblast and monocyte [26]. Circulating fibrocytes rapidly enter the wound site. Subsequently, TGF-*β*1 triggers the transdifferentiation of these cells into *α*SMA-positive myofibroblasts that express collagen type I, fibronectin, and vimentin and increase the amount and deposition of ECM components [98, 99]. Normally, circulating fibrocytes comprise less than 1% of all leukocyte populations, but during fibrotic changes derived from inflammation, the amount of these cells systematically increases [26]. Fibrocytes can be distinguished in at least 4-day-old skin wounds, and the quantity of these cells raises with time and increasing wound age [100]. For the differentiation of fibrocyte precursors, CD14-positive monocytes are stimulated through lymphocyte Th2-derived IL-4 and IL-13 [93]. The induction of fibrosis through fibrocytes is primarily based on the deposition of ECM components, as discussed above. Nevertheless, we cannot omit a variety of proinflammatory cytokines secreted from fibrocytes into the wound, namely, TNF-*α*, IL-6, IL-8, IL-10, and macrophage inflammatory protein 1*α*/*β* (MIP 1*α*/*β*) [27]. Moreover, it has been demonstrated that the fibrocytes in burned patients secrete TGF-*β*1, which activates myofibroblasts from existing fibroblasts [101] or triggers differentiation toward fibroblasts-like cells from surrounding endothelial or epithelial tissues through EndMT or EMT. However, due to dynamic nature of fibrocytes and constantly changing phenotype and functions of these cells, during their migration, some serious inconsistency appeared on the exact definition and identification of fibrocytes. These discrepancies are related to different methodology used to investigate fibrocytes involvement in variety of fibrotic disorders on variable stages. It has been suggested that one must categorize fibrocytes as functionally different depending on the isolation condition [102]. What is more, it is still unclear whether fibrocytes contribute only to worsening or improving tissue repair, as they possibly represent “the wrong cells in the wrong time” [102].

## 5. Concluding Remarks

**Figure 3:**
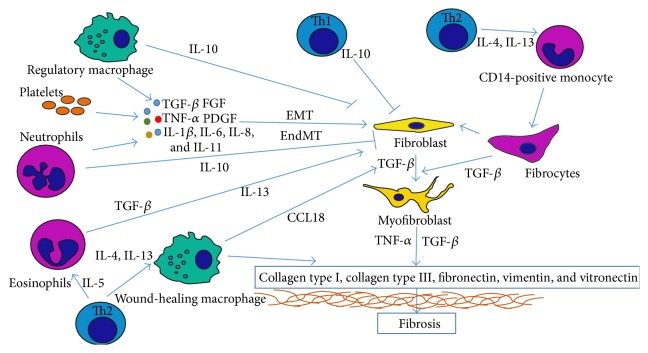
The comprehensive view on complex leukocytes impact on fibrotic tissue formation. The direct impact depends on ECM components production and deposition by leukocytes, such as fibrocytes and wound-healing macrophages. The indirect impact is composed of variety of different chemokines and growth factors interacting with cells, which in turn leads to ECM deposition.

Fibrosis is a complicated and composed process, leading to severe pathological disorders. The scarce formation of fibrotic tissue, comprising excess amounts of collagen type I, fibronectin, and other ECM components, deregulates normal tissue functions. Inflammation and inflammation-associated bone marrow leukocyte recruitment in wounded tissue trigger a cascade of events, leading to wound enclosure, scar formation, and, in case of prolonged inflammation, massive fibrosis. The impact of leukocytes on fibrosis formation might be generally divided into direct and indirect effects as shown in Figure 3. The direct impact is strongly associated with the production and excess deposition of ECM components. This effect is primarily observed with fibrocytes and alternatively activated, wound-healing macrophages. The indirect impact is far more complicated, as this effect is multistaged and associated with the activation and recruitment (including cells transdifferentiation and EMT/EndMT fibroblasts formation) of collagen-secreting cells, such as macrophages and myofibroblasts, and increased myofibroblast survivability through the downregulation of proapoptotic signals and increased inflammatory response times. All indirect profibrotic events occur through different chemokines or growth factors secreted from leukocytes. The most common, and likely, best-known indirect impact is correlated with TGF-*β* family proteins, as these molecules trigger both the endothelial and epithelial to mesenchymal transition and activate myofibroblasts from fibroblasts and fibrocytes, thereby increasing the production of ECM components. However, although this mechanism is well known, no antifibrotic therapy, based on TGF-*β* deactivation, has been implicated without disruption of the physiological function of this molecule. Several drugs for the downregulation of TGF-*β* transcription or signal transduction have been examined in the last stages of clinical trials [9]. Nevertheless, the impact of IL-6, IL-8, IL-4, IL-13, or TNF-*α* cannot be neglected. Th2-derived IL-4 and IL-13 are primarily responsible for macrophage collagen deposition and fibrocyte generation, whereas TNF-*α*, IL-6, and IL-8 are strongly associated with EMT and EndMT and myofibroblast survival and stimulation of collagen production. Thus, leukocyte interactions with wounded tissue cells and other leukocytes are extremely complicated and complex, bringing more questions than answers.

## Abbreviations

ALK2: Activin-like kinase 2
ALK5: Activin-like kinase 5
C5a: Complement component 5a
CD: Cluster of differentiation
CXCR1: Interleukin-8 receptor
ECM: Extracellular matrix
EMT: Epithelial to mesenchymal transition
EndMT: Endothelial to mesenchymal transition
FSP-1: Fibroblast specific protein 1
GSK-3: Glycogen synthase kinase 3
IFN-*γ*: Interferon gamma
IL: Interleukin
Mac-1: Integrin *α*M*β*2
MESECs: Mouse embryonic stem cell-derived endothelial cells
MIP 1*α*/*β*: Macrophage inflammatory protein 1*α*/*β*
MMPs: Matrix metalloproteinases
NF*κ*B: Nuclear factor kappa-light-chain-enhancer of activated B cells
PDGF: Platelet-derived growth factor
PECAM1: Platelet-endothelial cell adhesion molecule (cluster of differentiation 31, CD31)
RNS: Reactive nitrogen species
SM22*α*: Transgelin
SSc: Systemic sclerosis
TGF-*β*: Transforming growth factor
Th1/2: T helper cell 1/2
TIMPs: Tissue inhibitor of metalloproteinases
TNF-*α*: Tumor necrosis factor-*α*
T*β*RI: TGF-*β* receptor type I
T*β*RII: TGF-*β* receptor type II
*α*SMA: *α* smooth muscle actin.
